# The SKINT1-Like Gene Is Inactivated in Hominoids But Not in All Primate Species: Implications for the Origin of Dendritic Epidermal T Cells

**DOI:** 10.1371/journal.pone.0123258

**Published:** 2015-04-01

**Authors:** Rania Hassan Mohamed, Yoichi Sutoh, Yasushi Itoh, Noriyuki Otsuka, Yukiko Miyatake, Kazumasa Ogasawara, Masanori Kasahara

**Affiliations:** 1 Department of Pathology, Hokkaido University Graduate School of Medicine, Sapporo, Japan; 2 Department of Pathology, Shiga University of Medical Science, Otsu, Japan; Nazarbayev University, KAZAKHSTAN

## Abstract

Dendritic epidermal T cells, which express an invariant Vγ5Vδ1 T-cell receptor and account for 95% of all resident T cells in the mouse epidermis, play a critical role in skin immune surveillance. These γδ T cells are generated by positive selection in the fetal thymus, after which they migrate to the skin. The development of dendritic epidermal T cells is critically dependent on the *Skint1* gene expressed specifically in keratinocytes and thymic epithelial cells, suggesting an indispensable role for *Skint1* in the selection machinery for specific intraepithelial lymphocytes. Phylogenetically, rodents have functional SKINT1 molecules, but humans and chimpanzees have a SKINT1-like (*SKINT1L*) gene with multiple inactivating mutations. In the present study, we analyzed *SKINT1L* sequences in representative primate species and found that all hominoid species have a common inactivating mutation, but that Old World monkeys such as olive baboons, green monkeys, cynomolgus macaques and rhesus macaques have apparently functional SKINT1L sequences, indicating that *SKINT1L* was inactivated in a common ancestor of hominoids. Interestingly, the epidermis of cynomolgus macaques contained a population of dendritic-shaped γδ T cells expressing a semi-invariant Vγ10/Vδ1 T-cell receptor. However, this population of macaque T cells differed from rodent dendritic epidermal T cells in that their Vγ10/Vδ1 T-cell receptors displayed junctional diversity and expression of Vγ10 was not epidermis-specific. Therefore, macaques do not appear to have rodent-type dendritic epidermal T cells despite having apparently functional *SKINT1L*. Comprehensive bioinformatics analysis indicates that *SKINT1L* emerged in an ancestor of placental mammals but was inactivated or lost multiple times in mammalian evolution and that *Skint1* arose by gene duplication in a rodent lineage, suggesting that authentic dendritic epidermal T cells are presumably unique to rodents.

## Introduction

Dendritic epidermal T cells (DETCs) are a unique population of γδ T cells residing in the mouse epidermis, with a critical role in skin immunosurveillance, homeostasis and wound healing [1–4]. The majority of mouse DETCs express an invariant Vγ5Vδ1 T-cell receptor (TCR) [5] (using the nomenclature of Heilig and Tonegawa [6], also called Vγ3Vδ1 according to the nomenclature of Garman *et al*. [7]). It is believed that, through their invariant TCRs, DETCs recognize a limited set of “stress antigens” induced on damaged or dysregulated keratinocytes [3,8]. DETC progenitors develop in the fetal thymus as early as at embryonic day 14.5 through day 18 of gestation, after which they home to the epidermis [9,10].

Previous work showed that the epidermis of FVB/N mice from Taconic Farms (FVB/N Tac) virtually lacks canonical DETCs expressing Vγ5Vδ1 TCRs and harbors T cells expressing diverse γδ TCRs in a number comparable to that of other mouse strains [11]. Subsequently, this virtual absence of canonical Vγ5Vδ1 DETCs in FVB/N Tac mice was attributed to the inactivation of the *Skint1* (selection and upkeep of intraepithelial T-cells protein 1) gene, a gene expressed specifically in keratinocytes and medullary thymic epithelial cells at embryonic day 15 and continuing into adulthood [12]. SKINT1 protein is a membrane protein made up of an immunoglobulin variable (IgV) domain, an Ig constant (IgC) domain and three transmembrane domains (TMD) [12]. It is a member of the SKINT family, which in turn is a member of the butyrophilin family [13,14]. Although underlying molecular mechanisms are poorly understood, accumulating evidence indicates that the *Skint1* gene contributes to the selection, migration and development of DETCs expressing an invariant TCR [15,16].

In sharp contrast to rodents such as mice and rats [17], humans and chimpanzees have an inactivated SKINT1-like (*SKINT1L*) gene with multiple in-frame stop codons [12]. (The approved gene symbol for this human gene is *SKINTL*, but we call it *SKINT1L* for the sake of consistency in nomenclature in other mammalian species and to emphasize that it is a member of the *SKINT1* subfamily). Consistent with this, the human epidermis lacks a population of γδ T cells expressing an invariant TCR [18–20]. In the present study, we attempted to determine at which stage in primate evolution the *SKINT1L* gene was inactivated. Bioinformatics analysis of primate *SKINT1L* sequences revealed that the inactivation of *SKINT1L* took place in the hominoid lineage and that Old World monkeys (OWM) such as olive baboons, green monkeys, cynomolgus macaques and rhesus macaques retain intact *SKINT1L* genes. The epidermis of cynomolgus macaques, which were chosen as a representative of OWM, contained skin-resident γδ T cells expressing a semi-invariant TCR. However, these γδ T cells differed from rodent DETCs in that their TCR displayed junctional diversity and that the expression of the Vγ fragment they used was not epidermis-specific. Extensive analysis of available mammalian genome sequences indicates that *SKINT1L* emerged in an ancestor of placental mammals, but was inactivated or lost multiple times in mammalian evolution, and that *Skint1* arose by gene duplication in a rodent lineage, suggesting that DETCs are presumably unique to rodents.

## Materials and Methods

### Animal samples

Tissue samples were obtained from cynomolgus macaques also known as crab-eating macaques (*Macaca fascicularis*) maintained at Shiga University of Medical Science. Skin tissues were excised from the back or palm skin of healthy adult macaques after anesthesia or euthanasia. All other tissues were obtained from healthy adult female macaques after euthanasia. The thymus was obtained from a 100-day-old, dead male embryo. All animal experiments were conducted in strict accordance with the recommendations in the Guidelines for the Husbandry and Management of Laboratory Animals of Research Center for Animal Life Science at Shiga University of Medical Science and the Guidelines for the Care and Use of Laboratory Animals at Hokkaido University Graduate School of Medicine. Animals were singly housed in the cages equipped with bars for climbing and puzzle feeders for environmental enrichment under controlled conditions of light (12-h light/12-h dark cycle, lights on at 8:00 A.M.). Food pellets of CMK-2 (CLEA Japan, Inc., Tokyo, Japan) were provided once a day and drinking water was available *ad libitum*. The protocol involving cynomolgus macaques was approved by the Animal Experiment Committee of Shiga University of Medical Science (Permit number: 2012-10-1H). All surgery was performed under ketamine (5 mg/kg) and xylazine (1 mg/kg) anesthesia, and all efforts were made to minimize suffering.

### Database analyses

BLAST (BLASTN and TBLASTN) searches were performed against Ensembl and NCBI databases using human *SKINT1L* nucleotide (NR_026749.2) and mouse SKINT1 protein (NP_001096132.1) sequences as queries. Non-human primate species subjected to analysis were bushbaby (*Otolemur garnettii*), chimpanzee (*Pan troglodytes*), common baboon (*Papio hamadryas*), cynomolgus macaque, gibbon (*Nomascus leucogenys*), gorilla (*Gorilla gorilla gorilla*), green monkey (*Chlorocebus sabaeus*), marmoset (*Callithrix jacchus*), mouse lemur (*Microcebus murinus*), olive baboon (*Papio anubis*), orangutan (*Pongo abelii*), rhesus macaque (*Macaca mulatta*), squirrel monkey (*Saimiri boliviensis*) and tarsier (*Tarsius syrichta*). Protein domains were predicted using the SMART server (http://smart.embl-heidelberg.de/smart/set_mode.cgi). Signal peptides were predicted using the SignalP 4.1 Server (http://www.cbs.dtu.dk/services/SignalP/).

### Phylogenetic analysis

Amino acid sequences were aligned using the Clustal Omega or Clustal X program [21,22] with default parameters. The tree was constructed using the neighbor-joining algorithm implemented in the MEGA version 6.0 software [23]. The distance matrix was obtained by calculating p-distances for all pairs of sequences. Gaps were excluded using the pairwise-deletion option. The reliability of branching patterns was assessed by bootstrap analysis (1,000 replications).

### Isolation of cDNAs coding for cynomolgus macaque SKINT1L and expression analysis of cynomolgus macaque *SKINT1L* and TCR Vγ/Vδ gene segments

Total RNA was extracted from cynomolgus macaque thymus, kidney, heart, liver, lung, bladder, uterus, skin and epidermal lymphocytes using the RNeasy mini Kit (Qiagen GmbH, Hilden, Germany), following the instructions of the manufacturer. Purified RNA was then treated with RNase free-DNase (Invitrogen, Camarillo, CA) and converted to cDNA using SuperScript III RT polymerase (Invitrogen). *SKINT1L* cDNA was amplified from skin cDNA by 3'-rapid amplification of cDNA ends (RACE) using high fidelity polymerase chain reaction (PCR) polymerase KOD-Plus- (Toyobo, Osaka, Japan). The sequences of gene-specific primers, which were designed based on the genomic sequences retrieved from the NCBI database, were 5'-TTTGGTGTCACCTGGCTCAA-3' for the first round of PCR and 5'-TGGGACCATCTAGTTGCAGGAA-3' for nested PCR. PCR products were cloned into the pGEM-T easy vector (Promega, Madison, WI), and multiple clones were sequenced using an automated sequencer (the cynomolgus macaque *SKINT1L* cDNA sequence has been deposited in GenBank under accession number AB974689). To amplify cynomolgus macaque TCR Vγ/Vδ gene segments, cDNA from epidermal lymphocytes was used as a template. Primers used for amplification are listed in Table 1. The sequences of the primers used for amplifying junctional V(D)J sequences were as follows: 5'-ATTCGCCTTCTCGTCCTTCT-3' and 5'-TGACTTTTCTGGCACCGTTA-3' for Vγ10; 5'-GTGCATATTTGTGGCCTTCA-3' and 5'-ATTGACCAAGCTTGACAGCA-3' for Vδ1; and 5'-GTGGCCCAGAAGGTTACTCA-3' and 5'-CCTTCACCAGACAAGCAACA-3' for nested PCR amplification of Vδ1.

**Table 1:** Primers used for analyzing Vγ/Vδ gene usage^a^.

Primer	Sequence
Cγ reverse	5'-TGACTTTTCTGGCACCGTTA-3'
Vγ1 forward	5'-AGCACAAGGAAGAGCTGGAAT-3'
Vγ2 forward	5'- ATACACGCACAAGGTGGAGT-3'
Vγ3 forward	5'- TTGTTGAGTGGCACCTGAGT-3'
Vγ2/4 forward	5'-GATAGTTATGGAAGCACAAGGAACA-3'
Vγ9 forward	5'-CCTGCAGACATGCTGTCACT-3'
Vγ10 forward	5'-ATTCGCCTTCTCGTCCTTCT-3'
Vδ1 forward	5'-GTGGCCCAGAAGGTTACTCA-3'
Vδ1 reverse	5'-GGAGCTAGCTGCTTTCTGGA-3'
Vδ2 forward	5'-CTGTTGAGTTGGTGCCTGAA-3'
Vδ2 reverse	5'-CACCTTGGAAATTGTCCTTGA-3'
Vδ3 forward	5'-TTTCTACAGGGCAATGCTGT-3'
Vδ3 reverse	5'-GCTTCACAGAAAACCGTCCT-3'

^a^Vγ gene usage was evaluated using Vγ/Cγ primer pairs whereas Vδ gene usage was evaluated using pairs of primers designed within the Vδ gene segments.

### Immunohistochemistry

Skin tissues were harvested and embedded in paraffin after formalin fixation. 4 μm-thick paraffin sections were reacted with mouse anti-human TCR CγM1 monoclonal antibody (Ab) (γ3.20; Thermo Scientific, Waltham, MA) using an automatic stainer and visualized with 3,3'-diaminobenzidine. The sections were then counterstained with hematoxylin. For detecting CD3^+^ cells, rabbit anti-human CD3 polyclonal Ab (A0452; Dako, Glostrup, Denmark) was added for 1 h followed by incubation with Alexa Fluor 594-conjugated goat secondary Ab for 30 min at room temperature. The tissue sections were washed in PBS containing 0.1% Tween 20 and then mounted with Vectashield mounting medium (Vector Laboratories, Burlingame, CA). For double staining, the sections were stained first with mouse anti-human TCR CγM1 monoclonal Ab. After counterstaining and blocking with goat serum (100-fold dilution) for 1 h, they were stained with rabbit anti-human CD3 polyclonal Ab, and then mounted with VECTASHIELD mounting medium. Epidermal CD3^+^ γδ TCR^+^ cells were counted by analyzing serial sections of the cynomolgus macaque skin and their number was calculated per mm basement membrane using the ImageJ software (version 1.46r) [24]. The percentage of γδ TCR^+^ cells in CD3^+^ cells was scored in triplicate for each animal, and the data obtained from three animals were combined for statistical analysis.

### Isolation of epidermal lymphocytes from cynomolgus macaque skin

Epidermal lymphocytes were isolated as described [25] with some modifications. Briefly, subcutaneous fat was removed from macaque skin tissues with a razor blade. They were then cut into strips, and the epidermis was separated using 10 ml of RPMI 1640 containing 1 mg/ml of collagenase and 1 mg/ml of dispase II overnight at 37°C. Epidermal cell suspensions were filtered and enriched for lymphocytes using Ficoll-Paque gradient medium. After washing several times with PBS, cells were immediately frozen for subsequent RNA extraction.

## Results

### *SKINT1L* is inactivated in hominoids but is apparently functional in OWMs

**Fig 1:**
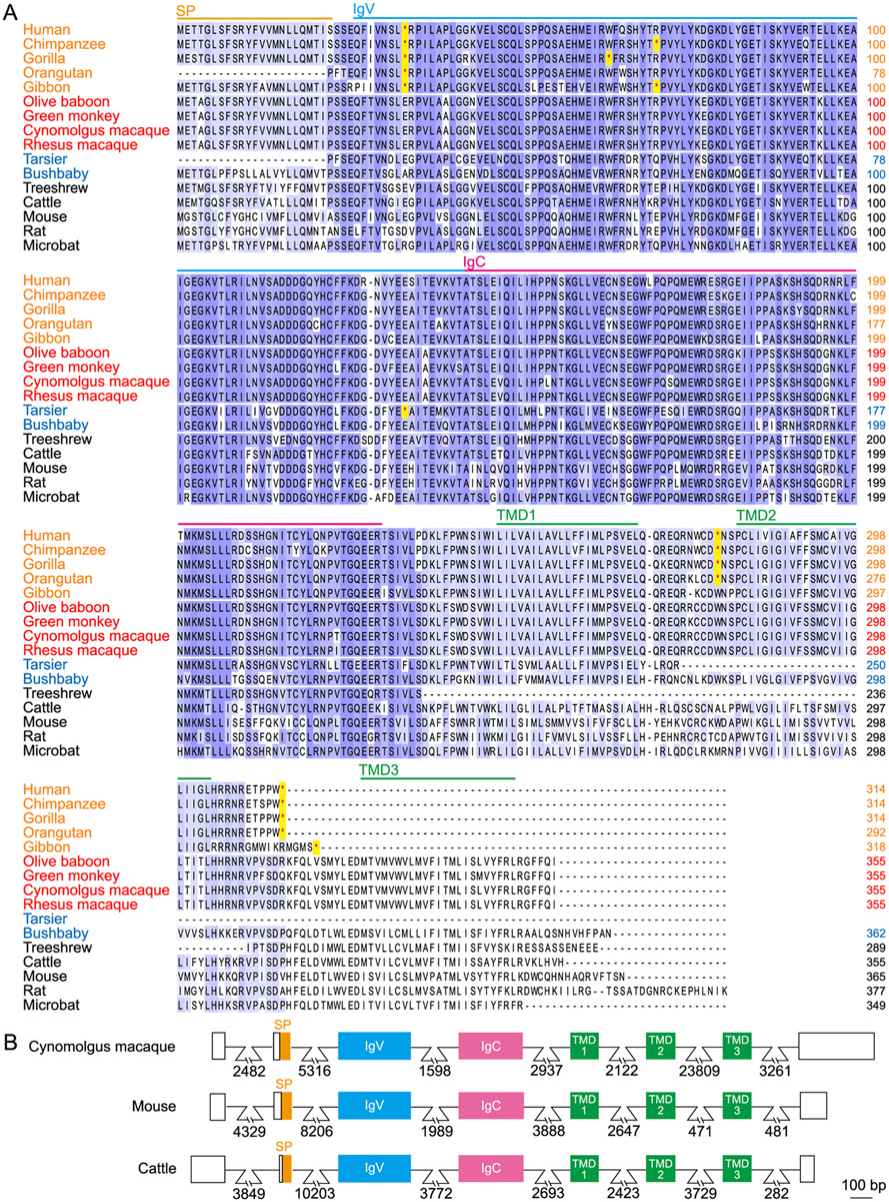
Amino acid sequence alignment of SKINT1 and SKINT1L molecules. A. Deduced SKINT1 and SKINT1L protein sequences of indicated species were aligned using the Clustal Omega program. Strictly and highly conserved residues are indicated in dark blue and light blue, respectively. Hominoid sequences contain multiple premature stop codons (indicated by red asterisks). The location of predicted domains is indicated on top of the sequences. Species names indicated in orange, red and blue represent hominoids, OWMs and prosimians, respectively. SP stands for signal peptides. Accession numbers are as follows: cattle, XP_005204826; microbat, XP_006107913.1; mouse, NP_001096132; rat, NP_001129388; and treeshrew, XP_006147332.1. Accession numbers and relevant information for primate sequences are given in S1 Table. B. The exon-intron organization of cynomolgus macaque *SKINT1L* was compared to that of mouse *Skint1* and bovine *SKINT1L*. Exon-intron boundaries were predicted based on the consensus splice junction sequences and similarity of the deduced amino acid sequences to the mouse SKINT1 protein sequence. Numbers indicate the length of introns in base pairs. Open boxes indicate 5'- and 3'-untranslated regions. bp, base pairs.

Human and chimpanzee *SKINT1L* genes are inactivated by multiple mutations, some of which are shared by both species [12]. To examine at which stage in primate evolution this inactivation took place, we analyzed *SKINT1L* sequences in primate species ranging from prosimians to hominoids (Fig 1A). This analysis, which involved human and 14 non-human primate species for which genome information is available, showed that all the hominoids including humans, great apes (chimpanzees, gorillas and orangutans) and lesser apes (gibbons) have *SKINT1L* genes inactivated by multiple mutations. One of the mutations, the stop codon located at the ninth residue of the IgV domain, was shared by all the hominoid sequences, suggesting that this mutation was responsible for the initial inactivation of the hominoid *SKINT1L* gene. Two mutations, one located between TMD1 and TMD2, and another located downstream of TMD2 leading to the elimination of TMD3, were shared by humans and great apes, but not by gibbons, suggesting that these mutations occurred in a common ancestor of humans and great apes after its divergence from lesser apes.

In sharp contrast, OWMs such as olive baboons, green monkeys, cynomolgus macaques and rhesus macaques have apparently intact SKINT1L sequences. Some prosimian species such as bushbabies also has apparently intact SKINT1L sequences. Furthermore, the SKINT1L molecules of OWMs and prosimians have three TMDs similar to rodent SKINT1 molecules. Therefore, we conclude that the stop codon at the ninth residue of the IgV domain shared by all the hominoid species took place in a common hominoid ancestor after its separation from OWMs. Interestingly, we could not identify SKINT1L sequences in the genome sequences of common baboons, marmosets, squirrel monkeys or mouse lemurs. It is possible that we failed to identify SKINT1L sequences in squirrel monkeys or mouse lemurs because their genomes have been sequenced only to low coverage.

### Cynomolgus macaques SKINT1L is expressed in the thymus and skin

**Fig 2:**
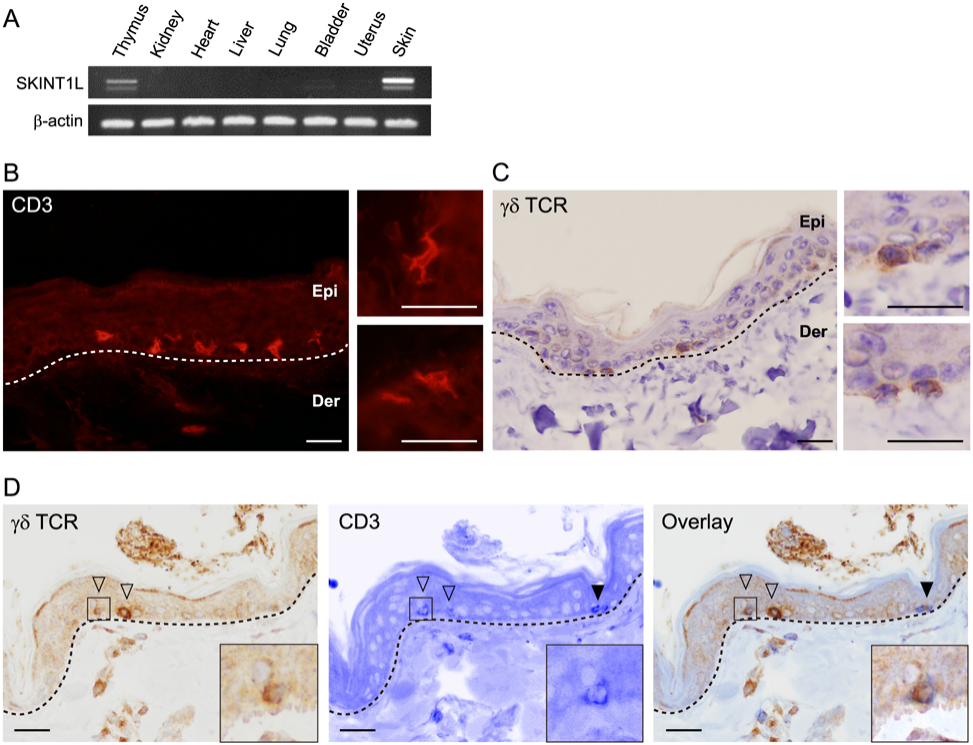
Tissue distribution of *SKINT1L* transcripts and immunohistochemical identification of DETC-like cells in the macaque skin. A. *SKINT1L* expression in cynomolgus macaque tissues was examined by reverse transcription PCR. β-actin was used as a control. Thymic tissues were obtained from a macaque fetus at embryonic day 100; the remaining tissues were procured from adult animals. A faint, lower band in the thymus and skin represents alternatively spliced transcripts encoding SKINT1L molecules with two TMDs. B-D: The basal and suprabasal layers of the epidermis of the cynomolgus macaque contain dendritic-shaped CD3^+^, γδ TCR^+^ cells. Paraffin sections of adult macaque skin were stained with cross-reacting antibody for human CD3 (B), cross-reacting antibody for human γδ TCR (C) or with both antibodies (D). Insets show higher magnification images of CD3^+^ dendritic-shaped cells (B), γδ TCR^+^ cells (C) and CD3/γδ TCR double-positive cells (D). Open and filled arrowheads indicate CD3^+^ γδ TCR^+^ cells and CD3^+^ γδ TCR^-^ cells, respectively. Scale bar, 10 μm. Epi, epidermis; Der, dermis.

We chose cynomolgus macaques as a representative of OWMs and isolated full-length SKINT1L-coding cDNA by 3'-RACE using skin cDNA as a template. Cloning and sequence analysis confirmed that the macaque *SKINT1L* gene is structurally intact as predicted by bioinformatics analysis and has exon-intron organization essentially identical to that of mouse and bovine *SKINT1L* genes (Fig 1B). The cynomolgus macaque genome contains only a single copy of the *SKINT1L* gene. Furthermore, *SKINT1L* is abundantly expressed only in the thymus and skin, suggesting that it might be the functional counterpart of mouse *Skint1* (Fig 2A). Sequence analysis of macaque *SKINT1L* transcripts revealed that the *SKINT1L* gene undergoes alternative splicing; besides the major transcript corresponding to the upper strong band in Fig 2A, we detected two splicing variants: one variant encodes SKINT1L molecules with only two TMRs (corresponding to a lower faint band in Fig 2A), and another variant produces transcripts that cannot encode functional SKINT1L protein because 38 base pairs immediately downstream of the slice donor site in intron 3 are not spliced out (the size of this transcript is indistinguishable from that of the major transcript in Fig 2A).

### Dendritic-shaped cells in the cynomolgus macaque skin

In mice, *Skint1* is required for the development of canonical Vγ5Vδ1^+^ DETCs and their homing to the epidermis [12,15]. To examine whether the cynomolgus macaque epidermis contains DETC-like cells, paraffin-embedded skin sections of three macaques were immunohistochemically stained with an Ab for human CD3, which detected CD3^+^ cells with a highly dendritic morphology in the basal and suprabasal layers of the epidermis (Fig 2B). On average, 4.2 ± 0.85 CD3^+^ cells were detected per mm of the basement membrane. When judged by double staining, approximately 41% of these CD3^+^ cells were stained with an Ab specific for the human TCR γ-chain constant region (1.7 ± 0.35 TCR γ-chain-positive cells per mm of the basement membrane) (Figs 2C and 2D).

**Fig 3:**
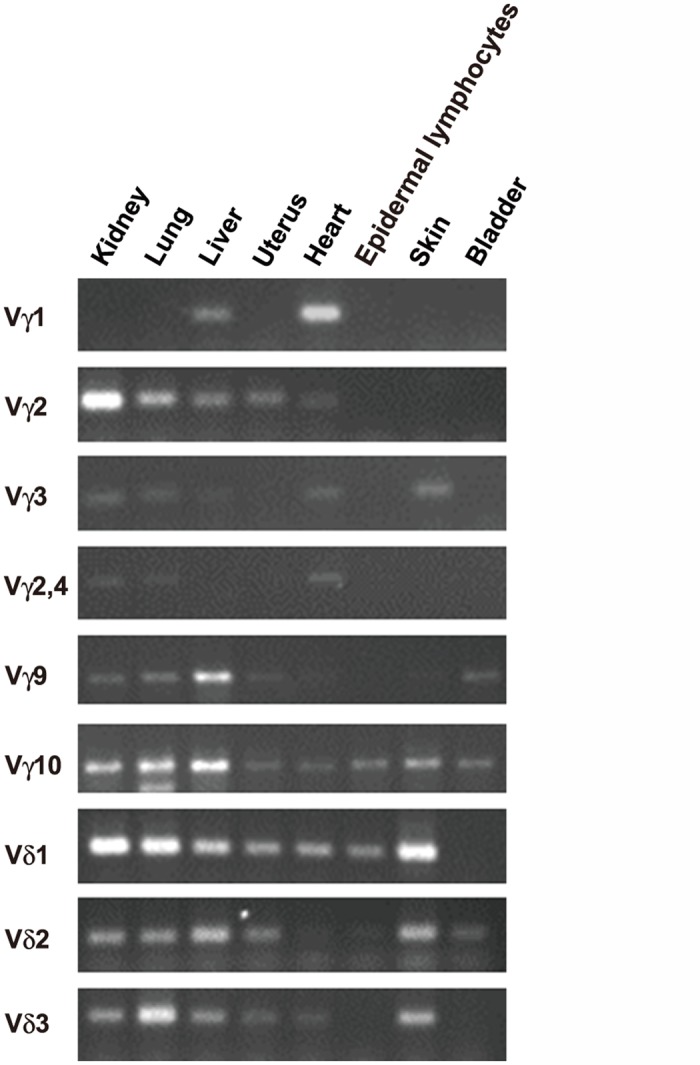
Cynomolgus macaque skin T cells predominantly express Vγ10Vδ1 TCR. A. Genomic organization of cynomolgus macaque TCR Vγ and Vδ loci. Green and yellow boxes indicate functional and non-functional gene segments, respectively. Numbers given in roman numerals over the blankets indicate the subgroups of Vγ gene segments defined by Huck *et al*. [27]. GenBank accession numbers for the macaque genomic sequences subjected to analysis were NC_022274 for the Vγ locus and NC_022278 for the Vδ locus. kbp, kilobase pairs. Arrows indicate orientation of transcription. B. Expression of cynomolgus macaque TCR Vγ and Vδ gene segments in representative tissues. Reverse transcription PCR was conducted using a set of primer pairs specific for each TCR Vγ or Vδ gene segment shown in Table 1.

To examine the nature of γδ TCR expressed by the CD3^+^ cells described above, we first analyzed the genomic organization of cynomolgus macaque TCR γ- and δ-chain loci (Fig 3A). The organization of the cynomolgus macaque TCR γ-chain locus was very similar to that of the rhesus macaque TCR γ-chain locus [26], and contained six functional Vγ gene segments: Vγ1, Vγ2, Vγ3, Vγ2/4, Vγ9 and Vγ10 (genes named according to Kazen *et al*. [26]). On the other hand, the TCR δ-chain locus contained three Vδ gene segments: Vδ1, Vδ2 and Vδ3 (Fig 3A).

To determine Vγ/Vδ gene usage in cynomolgus macaque epidermal T cells, we prepared a set of primers specific for each Vγ or Vδ gene segment. Because Vδ gene segments other than Vδ1–3 are used as Vα gene segments [28], we amplified only Vδ1–3 gene segments. Reverse transcription PCR analysis using these primer pairs showed that macaque epidermal T cells predominantly express Vγ10Vδ1 TCR (Fig 3B). However, unlike mice in which Vγ5 is expressed only in DETCs, macaque Vγ10 was also expressed in tissues other than the epidermis. As in human and mouse epidermis [11,29], Vδ1 was predominantly expressed in the macaque epidermis. Sequence comparison of mouse Vγ5 and human/macaque Vγ10 as well as that of mouse Vδ1 and human/macaque Vδ1 revealed only weak sequence similarity (S1 Fig). Phylogenetic analysis showed that macaque Vγ10 was not related to mouse Vγ5 and that macaque Vδ1 was not the counterpart of mouse Vδ1 (S2 Fig).

**Fig 4:**
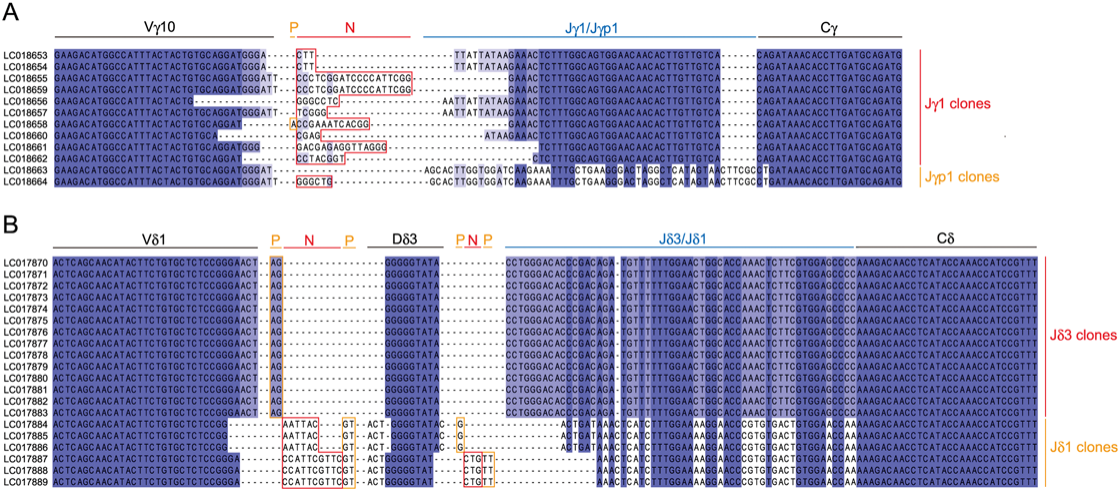
Junctional sequences of macaque TCR Vγ10- and Vδ1-chains. Panels A and B show the junctional sequences of TCR Vγ10- and Vδ1-chains, respectively. Strictly and highly conserved residues are indicated in dark blue and light blue, respectively. P- and N-nucleotides are boxed in orange and red, respectively. The sequences were deposited in GenBank under accession numbers LC018653-LC018664 (Vγ10-chains) and LC017870-LC017889 (Vδ1-chains).

To examine whether Vγ10Vδ1 TCRs expressed in epidermal lymphocytes are truly invariant, we amplified V(D)J junctional sequences from Vγ10- and Vδ1-chains by PCR. After cloning, multiple clones were sequenced for each chain (Fig 4). In the Vγ10-chain, two J segments, Jγ1 and JγP1, were used, and P- and N-nucleotides were present (Fig 4A). Of 12 clones subjected to sequencing, 10 had different sequences. Compared to the Vγ10-chain, the Vδ1-chain, which used either Jδ3 or Jδ1, showed much less diversity although P- and N-nucleotides were present; only three distinct sequences were isolated from 20 clones. Thus, unlike mouse DETCs, γδ TCRs expressed by macaque epidermal T cells were not invariant; however, four of the six complementarity-determining regions (CDRs), i.e., CDR1 and CDR2 of γ- and δ-chains, were invariant, and the CDR3 of the δ-chain showed limited diversity. These results indicate that macaque epidermal γδ T cells express a semi-invariant Vγ10Vδ1 TCR.

### Evolution of the *SKINT* gene family in mammals

Cattle have structurally intact SKINT1L molecules, and bovine skin γδ T cells predominantly express restricted sets of Vγ/Vδ gene segments (Vγ3, Vγ7, Jγ5, Cγ5 and Vδ sequences belonging to the Vδ1 family) [30,31]. To better understand the evolution of the *Skint1/SKINT1L* gene family, and more generally the entire *SKINT* gene family, we extended our bioinformatics analysis to mammals other than primates. A total of 47 species representing 22 orders, for which genome sequences are available, were subjected to analysis.

**Fig 5:**
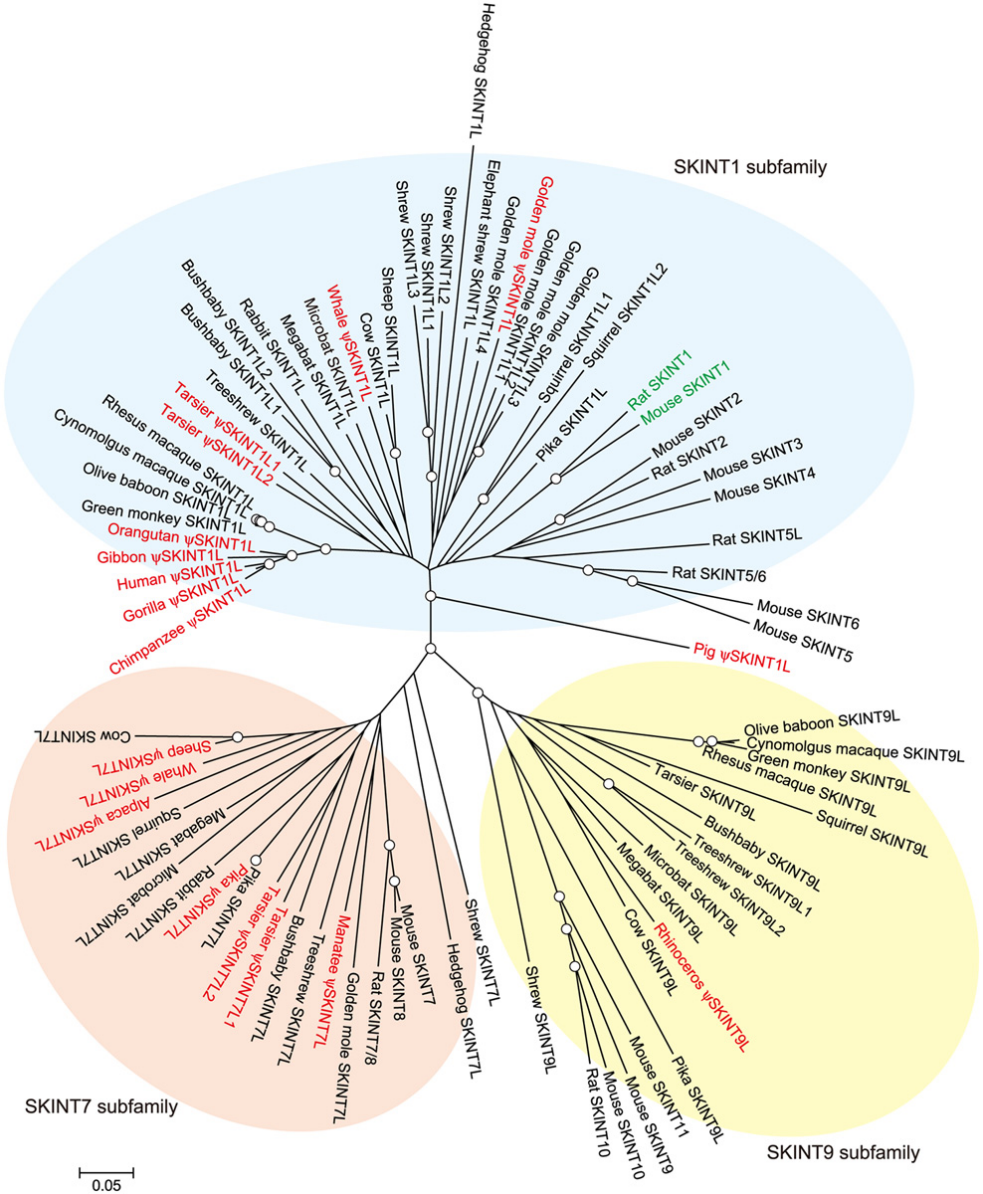
Phylogenetic tree of the SKINT family. The neighbor-joining tree was constructed based on deduced amino acid sequences as described in Materials and Methods. Nodes with bootstrap confidence values over 80 for 1,000 replications are indicated by circles. Genes judged to be non-functional are indicated in red. Genes proven to be functional are in green. Genes with no obvious inactivating mutations are in black. Accession numbers of sequences and other relevant information are given in S2 Table.

As shown previously [13], phylogenetic analysis based on the available amino acid sequences including IgV and IgC domains indicates that the SKINT protein family falls into three major subfamilies: SKINT1, SKINT7 and SKINT9 (Fig 5). SKINT2, 3, 4, 5 and 6 proteins are the members of the SKINT1 subfamily; they all appear to have emerged by rodent-specific gene duplication from the *Skint1* gene. Gene duplication is more extensive in mice than in rats, with mice having six and rats having three SKINT1 subfamily proteins. SKINT8 protein is closely related to SKINT7 protein and seems to have emerged by duplication from the *Skint7* gene in the mouse lineage. SKINT10 and 11 proteins are the members of the SKINT9 subfamily and appear to have emerged by rodent-specific gene duplication.

**Fig 6:**
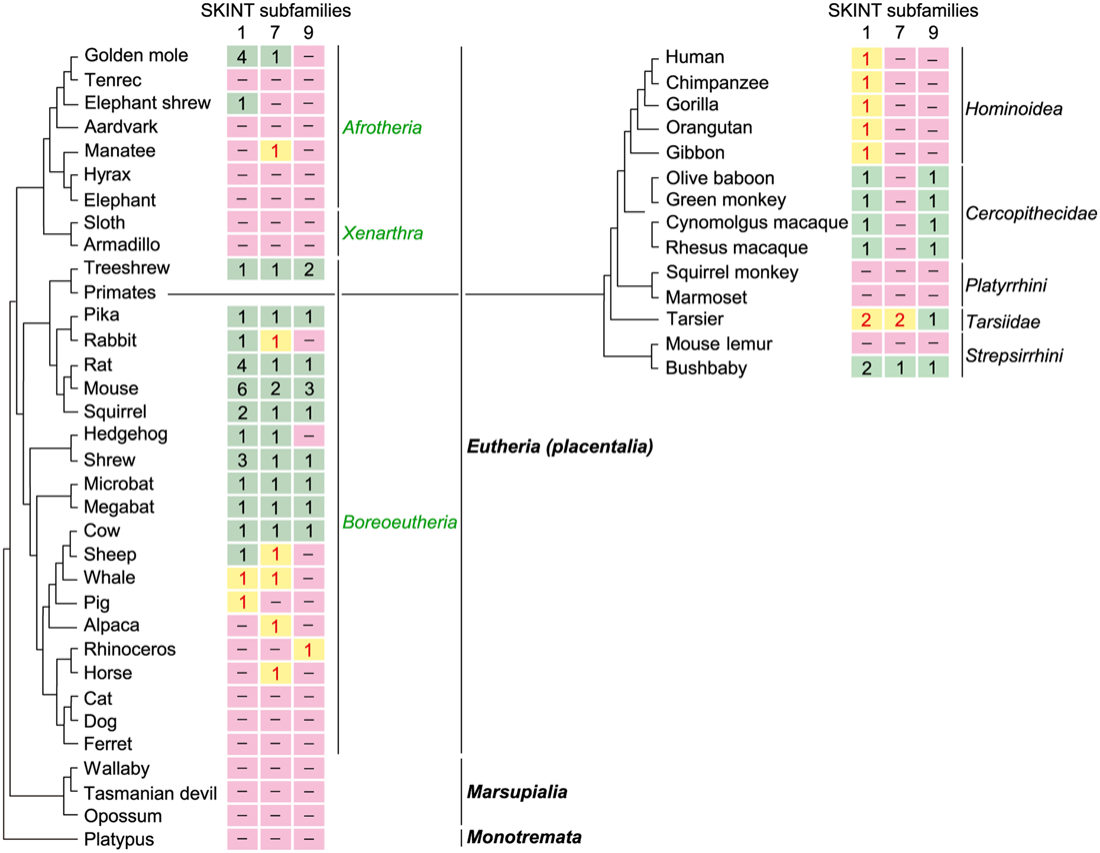
Distribution of SKINT subfamily proteins across mammalian orders. The phylogenetic relationship of mammals shown here is based on Murphy *et al*. [32] and Song *et al*. [33]. The copy number of *SKINT* genes belonging to each subfamily is shown for 47 mammalian species representing 22 orders. The copy number of functional and non-functional genes is indicated in black and red, respectively. The distribution of SKINT subfamily proteins in 14 primate species is shown separately on the right. Accession numbers of sequences and other relevant information are given in S2 Table.

Among mammals, SKINT family sequences were detected only in eutherian or placental mammals; neither marsupials nor monotremes had SKINT-like sequences (Fig 6). Furthermore, we were unable to identify SKINT-like sequences in non-mammalian vertebrates. Therefore, the SKINT family appears to have emerged in a common ancestor of placental mammals. The distribution of SKINT subfamily proteins across mammalian orders indicates that an ancestor of placental mammals had at least SKINT1 and SKINT7 subfamily proteins and that a boreoeutherian ancestor had all three subfamily proteins. A striking feature of the SKINT family is that its members were lost or rendered non-functional in many mammalian orders. Indeed, some mammals such as carnivorans appear to have lost the SKINT family in its entirety. The copy number of the *SKINT* gene family is increased in only a small number of species such as mice, rats, golden moles and treeshrews.

## Discussion

In the present study, we showed that all the hominoid species, for which genome sequence information is available, have a single copy of the *SKINT1L* gene and that it is inactivated by a shared stop codon (Fig 1). By contrast, the OWMs such as olive baboons, green monkeys, cynomolgus macaques and rhesus macaques have a single copy of structurally intact *SKINT1L*. Like its mouse counterpart, cynomolgus macaque *SKINT1L* is expressed in the thymus and skin, and the basal and suprabasal layers of the macaque epidermis contain a population of dendritic-shaped γδ T cells (Fig 2). Also, macaque epidermal T cells predominantly expressed Vγ10Vδ1 TCRs (Fig 3). These observations suggested that macaque *SKINT1L* might play a role equivalent or similar to that of mouse *Skint1*. However, contrary to our expectation, expression of macaque Vγ10 was not restricted to epidermal lymphocytes (Fig 3) and macaque Vγ10Vδ1 TCRs exhibited junctional diversity (Fig 4). This is in contrast to the situation in mice in which DETCs express an invariant Vγ5Vδ1 TCR without any junctional diversity and the Vγ5 gene segment is used exclusively in DETCs. Similar to cynomolgus macaques, cattle have a structurally intact SKINT1L molecule (Fig 1) [12]. Although it has been reported that bovine skin γδ T cells predominantly express restricted sets of Vγ/Vδ gene segments (Vγ3, Vγ7, Jγ5, Cγ5 and Vδ sequences belonging to the Vδ1 family) [31], the cattle epidermis does not appear to contain dendritic-shaped γδ T cells in a manner comparable to DETCs in rodents [30]. These results indicate that the presence of *SKINT1L* does not translate into the presence of rodent-type DETCs.

Because rodents have multiple paralogous copies of the *Skint1* subfamily, the *Skint1* genes of mice and rats are not orthologous to the *SKINT1L* genes of non-rodent mammals (Fig 5). Therefore, the *SKINT1L* gene might have a function distinct from that of the rodent *Skint1* gene and the emergence of DETCs might have been critically dependent on the emergence of the *Skint1* gene. Alternatively, *SKINT1L* might have a function equivalent or similar to that of rodent *Skint1*, but *SKINT1L/Skint1* alone might not be sufficient for the development of rodent-type DETCs. In mice, γδ T cells develop in discrete waves during fetal thymic ontogeny, with the first wave of γδ T cells giving rise to DETCs populating the epidermis [2,34]. Vγ5^+^ T cells are produced in the thymus only during a certain period of embryonic development and thereafter DETCs are renewed in the epidermis. The same V gene rearrangements and the junctional sequences (Vγ5Jγ1, Vδ1Dδ2Jδ2) for the first wave of γδ T cells are observed in the fetal thymus of TCR δ-deficient mice and wild-type mice [35], suggesting that these restricted γδ TCR rearrangements are not due to selection and hence are most likely *Skint1*-independent. In animals without this mode of γδ T cell development, it would be impossible to have rodent-type DETCs even if the *Skint1* gene is present. Similarly, if γδ T cells do not develop in discrete waves during the period when terminal deoxynucleotidyl transferase activities are absent, it would be impossible to have rodent-type DETCs expressing γδ TCRs without any junctional diversity. Therefore, animals such as macaques and cattle with structurally intact *SKINT1L* may lack rodent-type DETCs because they lack additional conditions required for the development of such cells.

The distribution of *SKINT1L* genes in representative orders of mammals including primates provides convincing evidence that an ancestral *SKINT1L* gene emerged in a common ancestor of placental mammals and that *SKINT1L* genes were lost in many orders of mammals (Fig 6). Interestingly, some prosimian species such as bushbabies has an apparently functional *SKINT1L* gene, but the genomes of NWMs such as marmosets and squirrel monkeys lack a sequence that qualifies as *SKINT1L* (Fig 6). Although the squirrel monkey genome has been sequenced only to low coverage, the marmoset genome is sequenced to high coverage. Thus, it seems likely that the *SKINT1L* gene was lost in marmosets. These findings suggest that SKINT1L function was lost at least twice in primate evolution, once by the elimination of the gene in some NWMs, and then by gene inactivation in a hominoid ancestor.

The distribution of SKINT subfamily proteins across mammalian orders shows that, similar to SKINT1L, members of the SKINT7 and SKINT9 subfamilies are frequently inactivated or lost (Fig 6). Indeed, only a limited number of species retain all of the three SKINT subfamilies, and some mammals such as carnivorans have completely lost the SKINT family. These observations indicate that the members of the *SKINT* gene family are generally dispensable and that they evolve in a highly order-specific or species-specific manner. Functional characterization of SKINT7 and SKINT9 proteins might help us understand why they are lost in some mammalian orders and whether animals without SKINT7 or SKINT9 have any compensatory mechanisms.

## Supporting Information

S1 Fig A. Amino acid sequence alignment of human Vγ10-, cynomolgus macaque Vγ10- and mouse Vγ5-chains. B. Amino acid sequence alignment of human Vδ1-, cynomolgus macaque Vδ1- and mouse Vδ1-chains. Sequences were aligned using the Clustal X program. Strictly and highly conserved residues are indicated in dark blue and light blue, respectively. GenBank accession numbers are as follows: human Vδ1, B32071; and mouse Vδ1, AAL08206. The remaining sequences were deduced from the genomic sequences: human Vγ10, NC_018918; cynomolgus macaque Vγ10, NC_022274; mouse Vγ5, NG_007033; and cynomolgus macaque Vδ1, NC_022278.
(TIF)

S2 Fig Phylogenetic trees of Vγ- and Vδ-chains. Phylogenetic trees were constructed as described in Materials and methods. Hs, *Homo sapiens*; Mf, *Macaca fascicularis*; and Mm, *Mus musculus*. GenBank accession numbers are as follows: HsVδ1, B32071; HsVδ2, CAA33277; HsVδ3, EAW66314; MmVδ1, AAL08206; MmVδ2–1, AAA84907; MmVδ2–2, AAL08208; and MmVδ4, AAL08209. Other sequences were deduced based on genomic sequences: MfVγ, NC_022274; MfVδ, NC_022278; HsVγ, NC_018918; and MmVγ, NG_007033.
(TIF)

S1 Table Accession numbers of primate SKINT1L sequences. (XLSX)

S2 Table Accession numbers of mammalian SKINT family genes. (XLSX)

## References

[1] Allison JP, Havran WL. The immunobiology of T cells with invariant γδ antigen receptors. Annu Rev Immunol. 1991; 9: 679–705. 1832874 10.1146/annurev.iy.09.040191.003335

[2] Hayday AC. γδ cells: a right time and a right place for a conserved third way of protection. Annu Rev Immunol. 2000; 18: 975–1026. 10837080 10.1146/annurev.immunol.18.1.975

[3] Witherden DA, Havran WL. Molecular aspects of epithelial γδ T cell regulation. Trends Immunol. 2011; 32: 265–271. 10.1016/j.it.2011.03.005 21481636 PMC3109268

[4] Yoshida S, Mohamed RH, Kajikawa M, Koizumi J, Tanaka M, Fugo K, et al Involvement of an NKG2D ligand H60c in epidermal dendritic T cell-mediated wound repair. J Immunol. 2012; 188: 3972–3979. 10.4049/jimmunol.1102886 22403443

[5] Itohara S, Farr AG, Lafaille JJ, Bonneville M, Takagaki Y, Haas W, et al Homing of a γδ thymocyte subset with homogeneous T-cell receptors to mucosal epithelia. Nature. 1990; 343: 754–757. 2154700 10.1038/343754a0

[6] Heilig JS, Tonegawa S. Diversity of murine gamma genes and expression in fetal and adult T lymphocytes. Nature. 1986; 322: 836–840. 2943999 10.1038/322836a0

[7] Garman RD, Doherty PJ, Raulet DH. Diversity, rearrangement, and expression of murine T cell γ genes. Cell. 1986; 45: 733–742. 3486721 10.1016/0092-8674(86)90787-7

[8] Hayday AC. γδ T cells and the lymphoid stress-surveillance response. Immunity. 2009; 31: 184–196. 10.1016/j.immuni.2009.08.006 19699170

[9] Xiong N, Kang C, Raulet DH. Positive selection of dendritic epidermal γδ T cell precursors in the fetal thymus determines expression of skin-homing receptors. Immunity. 2004; 21: 121–131. 15345225 10.1016/j.immuni.2004.06.008

[10] Xiong N, Raulet DH. Development and selection of γδ T cells. Immunol Rev. 2007; 215: 15–31. 17291276 10.1111/j.1600-065X.2006.00478.x

[11] Lewis JM, Girardi M, Roberts SJ, Barbee SD, Hayday AC, Tigelaar RE. Selection of the cutaneous intraepithelial γδ+ T cell repertoire by a thymic stromal determinant. Nat Immunol. 2006; 7: 843–850. 16829962 10.1038/ni1363

[12] Boyden LM, Lewis JM, Barbee SD, Bas A, Girardi M, Hayday AC, et al Skint1, the prototype of a newly identified immunoglobulin superfamily gene cluster, positively selects epidermal γδ T cells. Nat Genet. 2008; 40: 656–662. 10.1038/ng.108 18408721 PMC4167720

[13] Afrache H, Gouret P, Ainouche S, Pontarotti P, Olive D. The butyrophilin (BTN) gene family: from milk fat to the regulation of the immune response. Immunogenetics. 2012; 64: 781–794. 10.1007/s00251-012-0619-z 23000944

[14] Abeler-Dorner L, Swamy M, Williams G, Hayday AC, Bas A. Butyrophilins: an emerging family of immune regulators. Trends Immunol. 2012; 33: 34–41. 10.1016/j.it.2011.09.007 22030238

[15] Barbee SD, Woodward MJ, Turchinovich G, Mention JJ, Lewis JM, Boyden LM, et al Skint-1 is a highly specific, unique selecting component for epidermal T cells. Proc Natl Acad Sci USA. 2011; 108: 3330–3335. 10.1073/pnas.1010890108 21300860 PMC3044407

[16] Turchinovich G, Hayday AC. Skint-1 identifies a common molecular mechanism for the development of interferon-γ-secreting versus interleukin-17-secreting γδ T cells. Immunity. 2011; 35: 59–68. 10.1016/j.immuni.2011.04.018 21737317

[17] Kühnlein P, Mitnacht R, Torres-Nagel NE, Herrmann T, Elbe A, Hünig T. The canonical T cell receptor of dendritic epidermal γδ T cells is highly conserved between rats and mice. Eur J Immunol. 1996; 26: 3092–3097. 8977309 10.1002/eji.1830261240

[18] Foster CA, Yokozeki H, Rappersberger K, Koning F, Volc-Platzer B, Rieger A, et al Human epidermal T cells predominantly belong to the lineage expressing α/β T cell receptor. J Exp Med. 1990; 171: 997–1013. 2182763 10.1084/jem.171.4.997 PMC2187846

[19] Spetz AL, Strominger J, Groh-Spies V. T cell subsets in normal human epidermis. Am J Pathol. 1996; 149: 665–674. 8702004 PMC1865306

[20] Groh V, Porcelli S, Fabbi M, Lanier LL, Picker LJ, Anderson T, et al Human lymphocytes bearing T cell receptor γδ are phenotypically diverse and evenly distributed throughout the lymphoid system. J Exp Med. 1989; 169: 1277–1294. 2564416 10.1084/jem.169.4.1277 PMC2189233

[21] Sievers F, Wilm A, Dineen D, Gibson TJ, Karplus K, Li W, et al Fast, scalable generation of high-quality protein multiple sequence alignments using Clustal Omega. Mol Syst Biol. 2011; 7: 539 10.1038/msb.2011.75 21988835 PMC3261699

[22] Larkin MA, Blackshields G, Brown NP, Chenna R, McGettigan PA, McWilliam H, et al Clustal W and Clustal X version 2.0. Bioinformatics. 2007; 23: 2947–2948. 17846036 10.1093/bioinformatics/btm404

[23] Tamura K, Stecher G, Peterson D, Filipski A, Kumar S. MEGA6: Molecular evolutionary genetics analysis version 6.0. Mol Biol Evol. 2013; 30: 2725–2729. 10.1093/molbev/mst197 24132122 PMC3840312

[24] Schneider CA, Rasband WS, Eliceiri KW. NIH Image to ImageJ: 25 years of image analysis. Nat Methods. 2012; 9: 671–675. 22930834 10.1038/nmeth.2089 PMC5554542

[25] Schaerli P, Ebert L, Willimann K, Blaser A, Roos RS, Loetscher P, et al A skin-selective homing mechanism for human immune surveillance T cells. J Exp Med. 2004; 199: 1265–1275. 15123746 10.1084/jem.20032177 PMC2211907

[26] Kazen AR, Adams EJ. Evolution of the V, D, and J gene segments used in the primate γδ T-cell receptor reveals a dichotomy of conservation and diversity. Proc Natl Acad Sci USA. 2011; 108: E332–340. 10.1073/pnas.1105105108 21730193 PMC3141992

[27] Huck S, Dariavach P, Lefranc MP. Variable region genes in the human T-cell rearranging γ (TRG) locus: V-J junction and homology with the mouse genes. EMBO J. 1988; 7: 719–726. 2969332 10.1002/j.1460-2075.1988.tb02868.x PMC454380

[28] Klein MH, Concannon P, Everett M, Kim LD, Hunkapiller T, Hood L. Diversity and structure of human T-cell receptor α-chain variable region genes. Proc Natl Acad Sci USA. 1987; 84: 6884–6888. 3502713 10.1073/pnas.84.19.6884 PMC299189

[29] Ebert LM, Meuter S, Moser B. Homing and function of human skin gammadelta T cells and NK cells: relevance for tumor surveillance. J Immunol. 2006; 176: 4331–4336. 16547270 10.4049/jimmunol.176.7.4331

[30] Hein WR, Dudler L. TCR γδ+ cells are prominent in normal bovine skin and express a diverse repertoire of antigen receptors. Immunology. 1997; 91: 58–64. 9203966 10.1046/j.1365-2567.1997.00224.x PMC1364035

[31] Van Rhijn I, Rutten VP, Charleston B, Smits M, van Eden W, Koets AP. Massive, sustained γδ T cell migration from the bovine skin in vivo. J Leukoc Biol. 2007; 81: 968–973. 17234682 10.1189/jlb.0506331

[32] Murphy WJ, Pevzner PA, O'Brien SJ. Mammalian phylogenomics comes of age. Trends Genet. 2004; 20: 631–639. 15522459 10.1016/j.tig.2004.09.005

[33] Song S, Liu L, Edwards SV, Wu S. Resolving conflict in eutherian mammal phylogeny using phylogenomics and the multispecies coalescent model. Proc Natl Acad Sci USA. 2012; 109: 14942–14947. 10.1073/pnas.1211733109 22930817 PMC3443116

[34] Carding SR, Egan PJ. γδ T cells: functional plasticity and heterogeneity. Nat Rev Immunol. 2002; 2: 336–345. 12033739 10.1038/nri797

[35] Itohara S, Mombaerts P, Lafaille J, Iacomini J, Nelson A, Clarke AR, et al T cell receptor δ gene mutant mice: independent generation of αβ T cells and programmed rearrangements of γδ TCR genes. Cell. 1993; 72: 337–348. 8381716 10.1016/0092-8674(93)90112-4

