# Supplementary figures and images for: The SKINT1-Like Gene Is Inactivated in Hominoids But Not in All Primate Species: Implications for the Origin of Dendritic Epidermal T Cells
Source: PLoS One. 2015 Apr 1;10(4):e0123258. doi: 10.1371/journal.pone.0123258 (PMC4382165; doi:10.1371/journal.pone.0123258)

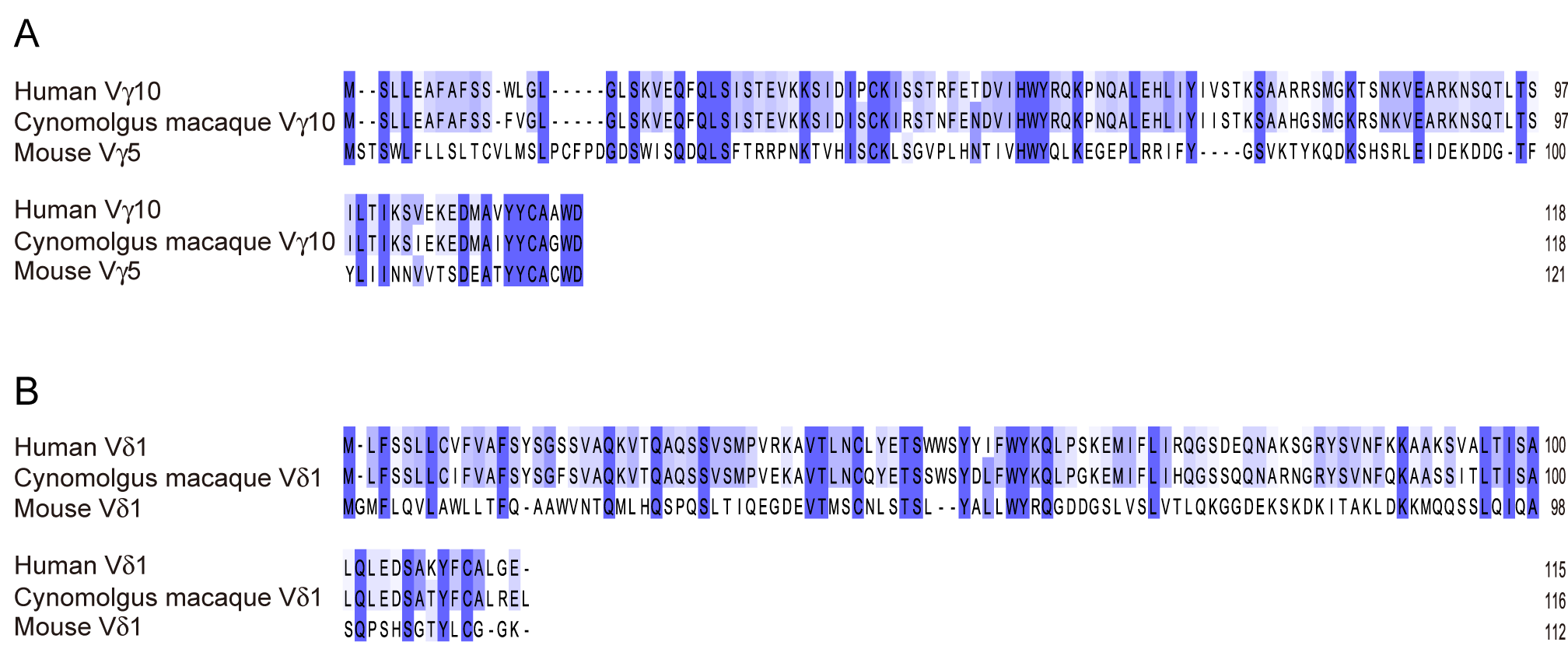

Supplement: S1 Fig — A. Amino acid sequence alignment of human Vγ10-, cynomolgus macaque Vγ10- and mouse Vγ5-chains. B. Amino acid sequence alignment of human Vδ1-, cynomolgus macaque Vδ1- and mouse Vδ1-chains. Sequences were aligned using the Clustal X program. Strictly and highly conserved residues are indicated in dark blue and light blue, respectively. GenBank accession numbers are as follows: human Vδ1, B32071; and mouse Vδ1, AAL08206. The remaining sequences were deduced from the genomic sequences: human Vγ10, NC_018918; cynomolgus macaque Vγ10, NC_022274; mouse Vγ5, NG_007033; and cynomolgus macaque Vδ1, NC_022278. (TIF) [file pone.0123258.s001.tif]

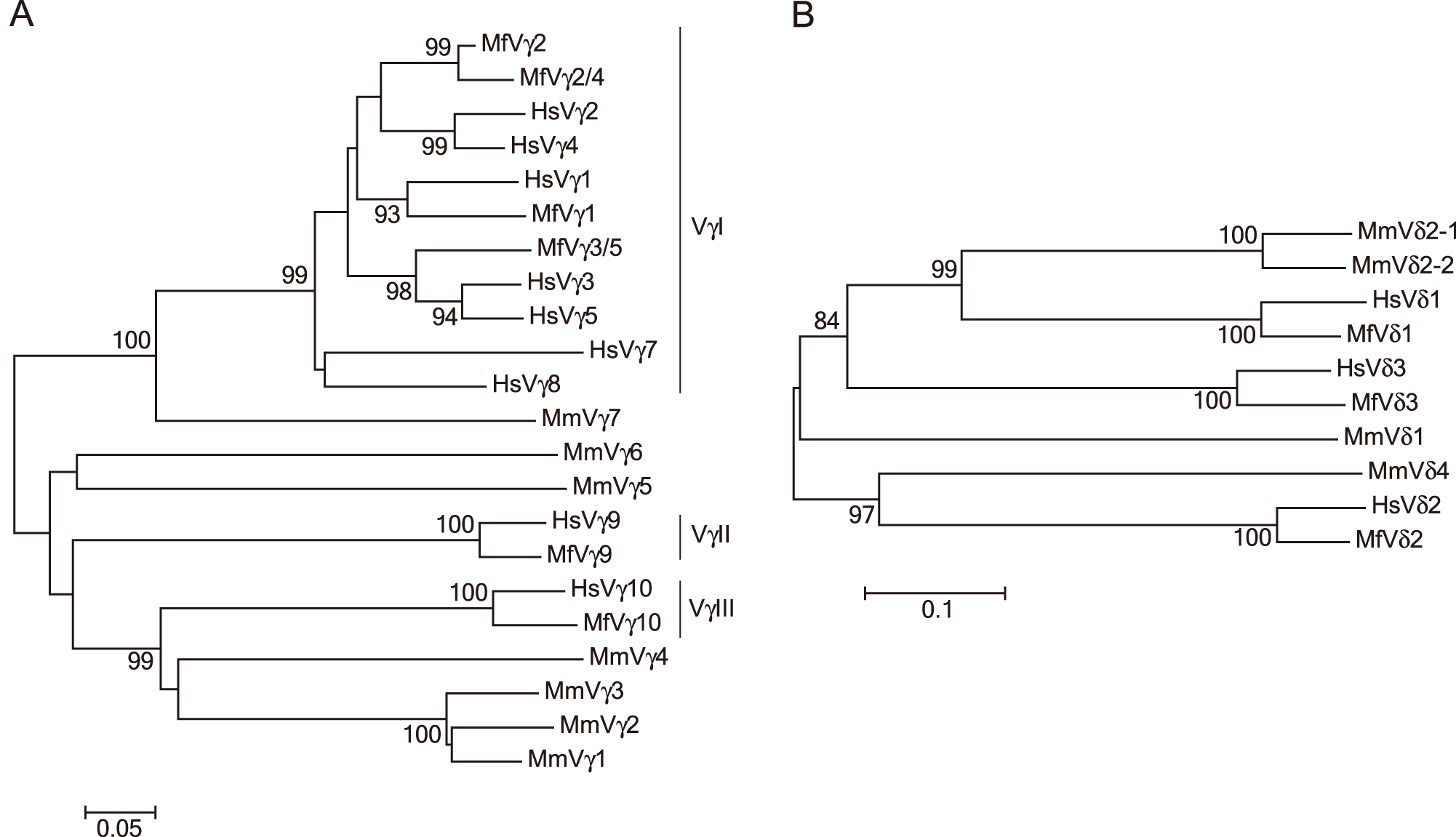

Supplement: S2 Fig — Phylogenetic trees were constructed as described in Materials and methods. Hs, Homo sapiens; Mf, Macaca fascicularis; and Mm, Mus musculus. GenBank accession numbers are as follows: HsVδ1, B32071; HsVδ2, CAA33277; HsVδ3, EAW66314; MmVδ1, AAL08206; MmVδ2–1, AAA84907; MmVδ2–2, AAL08208; and MmVδ4, AAL08209. Other sequences were deduced based on genomic sequences: MfVγ, NC_022274; MfVδ, NC_022278; HsVγ, NC_018918; and MmVγ, NG_007033. (TIF) [file pone.0123258.s002.tif]
